# Emotional Labour Behaviour of Nursing Students: A Phenomenological Qualitative Study

**DOI:** 10.1002/nop2.70362

**Published:** 2026-01-21

**Authors:** Dalyal Nader Al‐Osaimi

**Affiliations:** ^1^ Department Medical Surgical Nursing, College of Nursing King Saud University Riyadh Saudi Arabia

**Keywords:** education nursing baccalaureate, emotional labour behaviour, nursing practice, students nursing

## Abstract

**Aim:**

This study aimed to qualitatively explore the lived experiences of nursing students regarding their emotional labour behaviour and their encounters with managing feelings in healthcare settings.

**Design:**

A qualitative exploratory interpretive phenomenological design was conducted among 21 nursing students from different academic levels at one major university in Saudi Arabia.

**Methods:**

Three focus group discussions were carried out and thematic analysis was adopted to generate the findings. This study was reported using the COREQ Standards for Reporting Qualitative Research.

**Results:**

Inductive and deductive thematic analyses were used. The emergent themes were matched to the emotional labour conceptual framework. This resulted in three subthemes namely, “pretending”, “repression” and “distress” under the first main theme “surface acting”. The second theme “deep acting” reflected on professionalism and relationship of emotional labour to patient centered care. Finally, the third theme “genuine acting” had two subthemes; “favourable emotions” and “unfavourable emotions”. Students' interactions with patients and their effectiveness during clinical practice are both impacted by emotional labour behaviour. Receiving more education and training, as well as support from employed nurses and instructors can help students in controlling their emotions, thus safeguarding quality of care.

**Patient or Public Contribution:**

No Patient or Public Contribution.

**Abbreviation**
CLE: clinical learning environment

## 1 Introduction

A key element of the undergraduate nursing program is clinical practice. It happens in a setting known as the clinical learning environment (CLE), and research emphasises the difficulty of education in that kind of setting (Zhang et al. 2022). Previous literature argues that since patient care is prioritised over student instruction in hospital areas, the learning environment there is inherently more complicated than it is in the university setting (Zhang et al. 2022). The intricacy of acquiring knowledge in the clinical context is increased by issues that are common in the hospital setting such as difficult patients, nursing deficits, and intense workloads (Jamshidi et al. 2016). Additionally, most hospitals have insufficient resources and equipment for delivering medical care and are overcrowded with patients, which leaves less room for successful and enjoyable clinical training (Panda et al. 2021). These issues have a detrimental effect on clinical education and instruction. In turn, these stressful clinical practice experiences are expected to affect nursing students' emotional management, and thus affect their learning in a vicious cycle (Amoo et al. 2022).

## 2 Background

Hochschild, an American sociologist, was the one who originally introduced the idea of emotional labour. It can be boiled down to emotional management, which Hochschild establishes as the stimulation or inhibition of emotions in order to preserve an external embrace that generates the appropriate state of mind in other people, who are the subjects of care (Hochschild 1983). In her fundamental study on airline staff members, Hochschild asserts that smiling is an essential component of their profession, one that compels them to synchronise personality and emotion so that they would appear satisfied with their work (Hochschild 1983). She goes on to say that in order to increase passenger satisfaction, it is also part of the job to mask tiredness and annoyance. She implies that such an accomplishment necessitates emotional labour (Hochschild 1983).

Aviation attendants and nurses have common characteristics in that they are both absorbed in providing care in one form or another, and that stress may have a detrimental impact on the quality of the care they provide (Jongabb 2018). Nursing science recognises emotional labour as an integral component of the nursing role, as nurses frequently encounter emotionally challenging situations that require them to navigate their own emotions while attending to the emotional needs of others. These situations may include providing comfort to grieving individuals, offering support to anxious patients, or remaining composed during high‐stress situations (Henderson 2001; Kim 2020; Lee and Jang 2020). The emotional labour undertaken by nursing students is particularly relevant, as it influences their development as future healthcare professionals and has implications for their well‐being, job satisfaction, and ability to provide effective care (Karadaş et al. 2021).

Deep, surface, and genuine acting are the three components of the conceptual framework of emotional labour (Diefendorff et al. 2005). The capacity to truly and consciously experience the emotion that must be reflected at that precise moment through appropriate emotional adjustment and behaviour is known as deep acting. Surface acting is the process of someone merely altering their behaviour and behaving as though they are feeling something when they are not. Genuine acting is defined as acting in accordance with one's true feelings (Öz et al. 2023). As presented previously, emotional labour is especially evident in occupations that need frequent interaction with individuals, such as those in the medical field. In addition, in contrast with other workers in the service sector, nurses in the health sector must cope with a range of complex feelings (Kirk et al. 2021). While in frequent interaction with patients, nurses control their emotions and display emotional labour behaviour. Deep acting, can be highly advantageous for nurse practitioners as it involves genuinely experiencing and expressing emotions in a way that aligns with the desired emotional state. By engaging in deep acting, nurses can cultivate a sense of authenticity and sincerity in their interactions with patients, which may result in the creation of more positive emotional experiences. The act of genuinely empathising and connecting with patients can generate feelings of compassion, satisfaction, and fulfillment for nurse practitioners (Nassuna 2021). On the other hand, surface acting, may have detrimental effects on nurses. This can create a significant emotional burden for nurses, as they are forced to consistently portray emotions that may not reflect their true feelings (Nassuna 2021). Such incongruence between internal emotions and outward display can lead to increased stress, emotional exhaustion, and even burnout among healthcare professionals (Kim 2020). Studying the link among emotional labour behaviour and nursing care is a top concern, where the emotional labour behaviour of nurse practitioners is vital for ensuring that the patient feels secure (Doğan et al. 2022).

Teaching emotional labour behaviour in the undergraduate nursing program, to prospective nurses, is extremely important. For instance, previous research showed that it's crucial for student nurses to act appropriately and effectively control their emotions when they encounter a challenging circumstance in the hospital or when an individual passes away (Caminati et al. 2021). The incorporation of emotional labour behaviour in nursing school educational programs, relationships among students and educators in the hospital, and practical student nurses' conduct are all said to have a significant impact on emotional labour behaviour. Prior studies also showed that. Each phase of patient care involves emotional labour behaviour from nursing students, and uncontrollable emotions have a detrimental impact on the educational process (Baksi and Özer 2022). The greater the understanding of emotional labour behaviour from the outset of nursing school, the more positive an impact it will have on patient care, staff fulfillment, and healthcare administration. In order to assist students understand, accept, and effectively manage any feelings that they may encounter down the road, it is crucial to tackle their emotional experiences throughout clinical education prior to their entry into their actual professional practice (Anine et al. 2022). The literature is still nascent in examining emotional labour among nursing students, where only a few studies are published. In the Arab region no studies were found. Thus, the aim of this study is to qualitatively explore the lived experiences of nursing students regarding their emotional labour behaviour and their encounters with managing feelings in healthcare settings. The data will potentiate more pertinent investigations on nursing academia and nursing service administration. Further scientific investigation on the topic will also be advantageous, and information will be offered to direct future investigators.

## 3 Methods

### 3.1 Research Design

Through the utilisation of focus group discussions interviews with student nurses from various academic levels at one major nursing school in Saudi Arabia, this study employed a qualitative exploratory interpretive phenomenological approach. This aligns with the hermeneutic paradigm of phenomenology where the fore‐structures of the researcher are not bracketed but rather used in order to gain a deeper understanding of the phenomenon during analysis (Tuohy et al. 2013).

### 3.2 Sample

Participants in this study were nursing students enrolled in a prominent Saudi Arabian university's Bachelor of Nursing (BSN) program, spanning a range of academic levels. In order to be eligible for inclusion, a prospective participant must be 18 years old or older, enrolled in the BSN program, have attended at least one clinical course at the affiliated hospital, not have been enrolled in previous nursing programs such as technical nursing programs or bridging programs, not have previous professional experience in nursing, not have transferred to the program from a different field of study, and have provided informed consent. A purposive sampling technique with maximum variation sampling was employed to recruit an externally homogenous and internally heterogenous sample, thus capturing a wide range of perspectives. In this study, 21 students from a renowned university hospital in Saudi Arabia served as a purposive sample. An 84% response rate was achieved out of the 25 invited nurses, with the main barrier to engagement being the program workload. Of those 25, 21 responded and participated. To gather thorough and comprehensive data, students with a variety of age, gender, and academic levels were sought out (Table 1).

**TABLE 1:** Participant characteristics.

Variable	Category	*N*	%
Gender	Male	9	42.85
	Female	12	57.14
Academic level	First year	6	28.57
	Second year	3	14.29
	Third year	5	23.81
	Fourth year	7	33.33
Age (*M* ± SD)	21.59 ± 1.29		

Using Krueger and Casey's (2015) guidelines, which indicated that five to eight individuals in every session were sufficient, the total sample for the phenomenological research's focus group discussion was calculated. Student nurses at the selected university were informed of the focus group's objectives and procedures in detail before they participated. Three focus groups in all were held.

### 3.3 Setting

The institution addressed is a prestigious institution and was the first to open in Saudi Arabia. At this university, the nursing curriculum persists for four years and includes a required internship as a step toward entering the workforce. Students can choose from a variety of learning activities in the program, including high‐fidelity simulations, team‐based learning, problem‐based learning, video‐based simulations, and didactic lectures that take place in the classroom. Additionally, clinical practice at the associated hospital is made available to students and is expected of them during the study duration.

## 4 Procedure

### 4.1 Recruitment and Data Collection

A sampling list was required from the university's nursing program director, who was contacted. Nursing students who qualified were listed for the researchers' use. The students' institutional emails had been incorporated into the list. To 25 students who were chosen at random, an email invitation was extended. The email contained comprehensive study information, the lead investigator's contact information, and a permission form. Those who provided a completed consent form in response were enrolled. The two‐month data gathering period was from May 2023 to July 2023.

### 4.2 Interviews

The focus groups were overseen by an assistant professor of nursing from a separate university. One female investigator who had experience with focus groups and qualitative analysis conducted the interviews with the students. Participants were introduced to the investigator through the school faculty after receiving the email inviting them to join noting that the researcher had never met them before. The focus groups were conducted face to face which enabled the researcher to observe the dynamic of the group, body language, and take field notes and memos. Consulting the participants, an appropriate time frame for the conversations was chosen so that they could be present and offer accurate accounts of their interactions, especially given their busy timetables. To avoid the possibility of a moderator assuming control, the lead investigator and research assistant took turns moderating the talks. There were no repeat interviews and each focus group session lasted between 35 and 45 min. Focus group discussions with peers improved interaction because the supportive group dynamic encouraged knowledge flow (Patton 2014). To aid in the analysis of the data, field notes were made before, during, and after the discussions in order to record the researchers' impressions of facial expressions and the viewpoints of those who participated. The focus groups were audio recorded upon the consent of the participants in order to aid in transcription and analysis of the data. According to Krueger and Casey (2015)‘s questioning manual introductory questions should be straightforward for participants to reply to quickly and should not be questions about the topic of discourse. Instead, opening questions should identify the subject matter and give participants a chance to consider it briefly before speaking (Table 2). The following inquiries were collected from the source by Krueger and Casey (2015).

**TABLE 2:** Interview questions.

Introduction question	Can you introduce yourself to us and the group? What are your thoughts on the phrase “emotional labour”?
Transition question	What were your impressions on the first day of clinical practice versus now?
Key questions	How do you feel about your interaction with patients?
	How do you maintain a positive interaction with patients when you are emotionally well?
	How do you maintain a positive interaction with patients when you are emotionally in a bad place?
	How are your behaviours with patients influenced by your emotions?
	How do you manage those feelings?
	When faced with a challenging and unfair circumstance, how do you handle it?
Final prompt	Do you have anything further to say?
Probing questions	Could you give us a better description?
	Could you provide us with a better description?

### 4.3 Data Analysis

Inductive followed by deductive thematic analysis was used in the process of interpretive phenomenological analysis. The use of inductive thematic analysis involved identifying patterns, themes, and meanings that emerged directly from the data, without first being constrained by the pre‐existing conceptual framework. This approach allowed for the exploration of new and unexpected insights that may arise from the data. After that, deductive thematic analysis involved testing the pre‐existing conceptual framework against the data. By combining these two approaches within interpretive thematic analysis, the researcher aimed to enhance the rigour and depth of the analysis, as well as maintain the voice of the participants (Larkin et al. 2006). First, the ideas gathered from each session were quickly written down and printed. To verify accuracy, every transcription was checked against the audio that was obtained. The researchers listened to each recording several times and read through the scripts several times to ensure they had a good understanding of the material. Due to the fact that the talks were conducted in Arabic, the transcriptions of the interviews were given to two specialised professional translators who translated the transcripts both ways before sending them to an independent specialist for assessment. The main investigator and two other research assistants who are well‐rounded in qualitative analysis conducted the analysis to achieve analyst triangulation. To become acquainted with the information being analysed, interview materials were first reviewed and the interviews were converted into written language. Reading the transcripts of interviews and underlining pertinent or significant remarks helped us get more comfortable with the data. The verbal expressions were then read again, broken into smaller pieces, and their meanings were established using the primary investigator's viewpoint of phenomenological reduction for comparison with the original codes. Each of these categories was given an open‐ended coding treatment, and chosen statements were given names and placed in categories based on how similar or dissimilar the encodes were; thus inductive thematic analysis was undertaken. Using deductive analysis, those subthemes were tested against the emotional labour conceptual framework and matched to the three levels of acting: surface, deep, and genuine. All codings were reviewed and again assessed by the third assistant, who was not involved in the initial coding procedure. The final themes and sub‐themes were decided upon after multiple revisions.

### 4.4 Trustworthiness and Credibility

To improve the study's credibility and guard against biases, the researchers employed a range of techniques in line with prior qualitative research (Lincoln and Guba 1986). Everyone who participated gave their consent to take part in the research, and the investigator, who was not related to them personally or professionally, stressed that there were no right or wrong responses and urged them to express their opinions. As a result, the discussions' credibility was guaranteed. The interviewer received training in qualitative investigation. The findings have gained credibility because of the investigator's deep engagement with the subject matter and immersive communication with the participants. The researcher spent around two months working on the task at hand as a consequence. To determine if the conclusions drawn from the data were compatible with the respondents' opinions, member checking was used. Conformability was evaluated by an impartial reviewer with expertise in qualitative research. The generated findings were submitted to peers for a third‐party review, their appropriateness was looked into and confirmed, and consensus was obtained. Additionally, the transferability and dependability of the data were improved by the purposive sampling strategy, which assisted in gathering a varied sample. The researchers released the whole study procedure, which enabled others to do follow‐up research, in order to assure dependability. All focus groups used the same in‐depth questions, and the investigator and assistants made sure that any novel concepts were thoroughly probed to remove any data blind spots. The participants in the study were given a true voice via the use of different sentences from the verbatim to sum up the findings (Speziale et al. 2011).

### 4.5 Ethical Considerations

The researchers were given approval to conduct the study by the university's Research and Ethics Committee (Eco‐r‐180). The objectives of the study had previously been explained to participants, who had also signed an official statement of consent to take part. This project was completed in accordance with the global Declaration of Helsinki's objectives and requirements. The transcripts were tagged and anonymised, and every individual was assigned a pseudonym, in order to safeguard their identities. The linguistic experts were given the transcriptions in a sealed package, and they gave it back to them in a sealed package. The focus group tapes were kept in an encrypted folder and were inaccessible to everyone.

## 5 Results

Upon conducting thematic analysis, the results inductively gave rise to five sub‐themes, which later deductively were matched to the dimensions of the emotional labour behaviour concept. Under the “surface acting” theme, three subthemes emerged namely, “pretending”, “repression”, and “distress”. Under the “deep acting theme” one subtheme emerged which is “professionalism”, and under the genuine acting theme two subthemes emerged, namely, “favourable emotions” and “unfavourable emotions” (Table 3).

**TABLE 3:** Thematic tree.

Themes	Subthemes	Quotes
Surface acting	Pretending	“…*I frequently found myself pretending to get through psychologically challenging circumstances…*”
		“…*We are frequently urged to smile and be upbeat despite whatever personal difficulties…*”
	Repression	“*…I thus hide my emotions to match the part…*”
	Distress	“*…I couldn't help but feel so upset after seeing a patient's severe state…*”
		“*…I feel so frustrated and upset when I take harsh comments from some of the nurses…*”
Deep acting	Professionalism	“*…but I feel like I'm contributing significantly to my patients' treatment when I can sympathize with them and actually understand their feelings…*”
Genuine acting	Favourable emotions	“*…Their pleasure made my day, to be sure…*”
	Unfavourable emotions	“*…When they needed it, I sobbed alongside them and lent a sympathetic ear…*”

### 5.1 Surface Acting

#### 5.1.1 Pretending

The study discovered that among nursing students, emotional labour strategies such as surface acting are often used. Several respondents acknowledged that they acted superficially when speaking with patients and coworkers. There were several factors shown to be the catalysts for nursing students to act superficially. These factors made it tough for students to authentically understand and express their feelings, and those comprised a heavy workload, time restraints, a lack of encouragement, and emotionally trying circumstances. The results showed a mismatch between the feelings nursing students had on the inside and the emotions they showed outwardly. Students frequently expressed feeling tension, irritation, or tiredness on the inside while projecting empathy, caring, and optimism on the outside as would be anticipated of them in their job. For instance, one student said, “During my clinical training, I frequently found myself pretending to get through psychologically challenging circumstances. There were times when I felt anxious and overburdened, but I had to maintain my composure and sympathise with the patients. You understand, it's like putting on a mask. Even though, deep inside, you're experiencing something different, you have to grin and seem like all is OK.” *(S7)* Another student also shared a similar account, “I think that in our line of work, pretending is fairly unavoidable. We have so much to cope with every day that we occasionally lack the time to examine our own feelings. I recall this one instance where I was dealing with a personal matter but had to keep it under wraps throughout my shift. When you're emotionally disconnected from your patients, it's difficult to connect with them on a true level…so you pretend…you put a smile, provide care and when you exit the room you get bombarded with all the emotions you have…” *(S19)*.

The impact of pretending on nursing students' emotional health was considerable. Due to the stress of consistently expressing feelings that did not correspond with their genuine sentiments, a number of respondents stated that they felt burned out and mentally distant from their job. The study focused on the impact of the hospital environment on the use of surface acting. Some individuals said that the workplace expectations of repressing personal feelings and focusing on emotional presentation during training led to pretending as a way to cope. For example, one student said, “We are frequently urged to smile and be upbeat despite whatever personal difficulties we may be experiencing. It's as though we've been trained to put the patients' feelings before our own. We are ignoring our mental health, which I believe might be bad in the long term…” *(S2)*.

In comparison to younger students, older nursing students, who were often in their final years of nursing school, had more emotional development and a sense of self. They appeared to be more capable of handling the emotional demands of patient care as a consequence, perhaps lowering the requirement for pretending. On the other hand, because they are still honing their emotional control abilities, newer nursing students, particularly those in their early academic years, were more likely to depend on pretending. For instance, one student said, “…I'm growing better at establishing deeper connections with patients as I get more expertise in patient care. I've learnt to control my emotions and, wherever possible, refrain from pretending. I believe it is crucial for me to be genuine in all of my contacts, and I've found that patients value this authenticity…” *(S15)*.

#### 5.1.2 Repression

The study found that many nursing students admitted to repressing their emotions throughout the course of their interactions and practical experiences. Repression entailed purposefully repressing or hiding their genuine emotional reactions, particularly when confronted with difficult or upsetting circumstances. The results showed that some nursing students suppressed their feelings because of a concern of being weak or vulnerable in front of patients, teachers, or peers. They thought that showing their actual feelings may have a detrimental effect on their professional image or be viewed as unprofessional. For instance, one of the students shared, “…It's difficult to display weakness, especially if you're the one who should be the capable caregiver. I do, however, occasionally wish I could communicate my worries and anxieties without fear of criticism…” *(S17)*. Some nursing students understood that suppressing their emotions can compromise the effectiveness of patient care and the therapeutic alliance. They found it more difficult to truly care for their patients and felt less connected to them as a result of suppressing their feelings. For example one of the testimonials was: “I struggle with wanting to provide compassionate care and feeling under constant pressure to maintain my composure. I thus hide my emotions to match the part. However, it is draining, and I worry that it could be harming how well I interact with my patients…I feel like I look cold with my patients…” *(S21)*. Another student said, “…I tend to focus on the procedures rather than the patient so that I won't have to show any emotion…Otherwise I break…So I keep looking at the paper in my hand or I tend not to talk much with the patient because I am not feeling well…” *(S12)*.

#### 5.1.3 Distress

The participants also reported that emotional overload affects them often, especially in stressful clinical settings or while dealing with emotionally taxing patient situations. As students struggle to control their own emotions while caring for others, this emotional weight may cause anxiety and discomfort. After seeing horrific occurrences like patient deaths or serious emergencies, some nursing students said they became agitated and distressed. Students may find it difficult to adequately manage their emotions after seeing such incidents since it might be emotionally exhausting. For instance, one student proclaimed: “I couldn't help but feel so upset after seeing a patient's severe state. Throughout the shift, I made an effort to remain composed, but it proved difficult so I broke in tears in front of everyone. It seems like these experiences have burdened me emotionally, and it is difficult for me to let go…” *(S1)*. Another student also said, “I come across emotionally difficult instances during rotations in the hospital that stay with me long after my shift. My distress persists, and it is difficult for me to relax. I wished there were more chances to reflect and work through these feelings…” *(S13)*. The participants also mentioned how being criticised by nurses and instructors made them more agitated and distressed. Their emotional load was compounded by unfavourable comments or critical remarks, making it harder for them to handle the demanding emotional needs of patient care. Nursing students' sentiments of inadequacy were reinforced by criticism from more seasoned nurses or teachers, which made them more distressed and agitated. One shared anecdote was, “…I can't help but feel so frustrated and upset when I take harsh comments from some of the nurses. I wonder if I'm cut out for this since it feels like my attempts aren't sufficient. More positive reinforcement would be nice, especially at trying times…” *(S18)*. Another student also said, “…It is extremely unsettling to be under teachers' and nurses' continual watch. I get a wave of anxiety every time I make a mistake because I am worried about how they'll respond. I try to keep my feelings to myself and put on a happy face, but on the inside, I'm really battling…” *(S8)*.

### 5.2 Deep Acting—Professionalism

The participants indicated that deep acting increased the degree of empathy and patient‐centered care displayed by them. These students were better able to deliver kind and tailored treatment because they were able to connect with patients' emotions and comprehend their viewpoints. Contrary to surface acting, deep acting was linked to lower emotional tiredness levels. Nursing students were more grounded and motivated in their caring roles by giving themselves permission to openly feel and express their emotions. For instance, one student said, “It's not always simple, but I feel like I'm contributing significantly to my patients' treatment when I can sympathize with them and actually understand their feelings…” *(S15)*. Another student also proclaimed, “…I can better handle the highs and lows of patient care when I honestly feel and process my emotions. It has to do with acting honestly in my capacity as a nurse…”*(S6)*. As shown the students related deep acting and processing of emotions to their role as a professional nurse. Another testimonial was, “…*Being compassionate has altered my viewpoint. I used to believe that controlling my emotions was the best way to be professional. By communicating the emotion I feel from the patient at the appropriate moment and in the appropriate context, depending on the circumstance, I may improve patient care…that is my role at the end of the day…*” *(S3)*.

### 5.3 Genuine Acting

#### 5.3.1 Favourable Emotions

When practicing authentic acting, participants reported feeling a sense of personal satisfaction and professional pleasure. Knowing that their positive genuine expressions of emotion had a beneficial influence on patients and improved their wellbeing, they found significance in their work as caregivers. A student said: “…I constantly try to encourage my patients to share in the joy of even the tiniest successes. We recently celebrated with a patient after they finished a difficult treatment session. Their pleasure made my day, to be sure…” *(S11). Another student also said*, “…I frequently utilized comedy in pediatrics to reduce anxiety before surgeries. It was remarkable to witness how a simple joke or amusing expression could change the child's tears into laughing and lessen the anxiety of the situation…” *(S5)*. A helpful and encouraging work atmosphere for some of the nursing students and their coworkers was made possible through genuine acting. The sincerity of emotional reactions encouraged open dialogue and teamwork among the medical staff. One quote that stood out from the rest was, “…*I also discovered how crucial it is to be honest about my feelings with my coworkers. When I was feeling overburdened with work one day, I asked for assistance rather than acting as though I had everything under control. My nursing colleagues were kind and helpful…*” *(S1)*.

#### 5.3.2 Unfavourable Emotions

The participating students not only highlighted being genuine about their positive emotions but also about their negative ones. They indicated that even if they were not feeling well, they felt like they were lying to their patients and colleagues if they hid their feelings. One student said, “…*Before a surgery, I had a patient who was really worried. I sat with her and discussed my personal experiences with comparable surgeries rather than only assuring her that everything would be great. It was wonderful to connect with her authentically, and I could see the sense of relief in her eyes… I felt the accomplishment…*” *(S14)*. Another student said, “…I experienced a range of emotions when providing end‐of‐life care, but I was aware that I had to be honest with the patient's family. When they needed it, I sobbed alongside them and lent a sympathetic ear. Although it was challenging, they eventually thanked me for supporting them during this trying period…when the patient died I felt like I failed him and shared those feelings and sought comfort…” *(S20)*.

## 6 Discussion

The present research, which examined nursing students' encounters with emotional labour behaviour, found that all of the participants had encountered such behaviour. Similar to other research, emotional labour actions were shown to be prevalent among students studying nursing (Baksi and Özer 2022; Ha et al. 2021). In the context of the surface acting theme, it was found that during the discussions with students' nurses, the students expressed that they were pretending to be happy while still working, regulated their anger while working, did not express their angry feelings to care recipients, and concealed their feelings because they believed the patient was not to blame. Students reported that the further they kept receiving their education, the more at ease attitudes they demonstrated in clinical settings. Initially, they admitted that they were reluctant and even surprised while speaking with clients. This result from this study is significant because it demonstrates how clinical procedures cause emotional labour behaviour in trainees. In research studies tackling the experiences of students, it was found that the anxiety and tension levels were highest prior to practicing clinically, and that students who had previously engaged in clinical work encountered a comparable amount of stress when beginning a new work experience, and that they had fears regarding clinical practice (Wu et al. 2021; Liu et al. 2022; Andrew et al. 2022). In addition, the participants said that the instruction and coursework they took improved their conduct, and that as their educational level rose, so did the manners with which they interacted with patients. This demonstrates how training and expertise may lessen the need for surface acting. Due to their elevated level of integration with respect to the actions and interactions necessary for the line of work, as well as the fact that their life knowledge grows with age, it is claimed that staff members who obtain sufficient instruction pertaining to their profession are better able to regulate their emotions and to be more effective in the task of displaying emotional labour (Yordanova and Dineva 2022). This is consistent with previous research, where it has been shown that there is not a disparity in emotional labour between different academic years (Karadaş et al. 2021) but that there is a negative correlation between age and deep and authentic acting that was substantiated by previous studies. Additionally, it was discovered that participants exhibited more surface behaviour in clinical settings when they weren't receiving support from the senior nurses. According to a previous study, emotional labour behaviour and clinical stress encountered by healthcare workers are negatively correlated (Chen et al. 2023). Uncontrollable feelings have a detrimental impact on the students' educational process, as students exhibit emotional labour behaviour throughout each step of helping patients. Additionally, it suggests that the relationship between pupils and instructors in the hospital as well as the conduct of clinical nurses with students are significant factors in emotional labour behaviour (Rozario et al. 2022). Instructing students in the hospital is also described as a task full of sentiments. In addition, when it came to the deep acting concept, a large number of participants said that they attempted to be optimistic toward patients, that they enjoyed their jobs as nurse practitioners that they made an effort not to show their unfavourable feelings, that they initially behaved in a nonprofessional manner but began to do so after they realised their errors, that they attempted to employ the proper communication methods to safeguard the confidentiality of patients. Previous work indicated that deep acting was the most frequently noticed in research involving student nurses and professional nurses (Theodosius et al. 2021; Lee and Jang 2020).

The participants mentioned that deep acting enabled them to be more empathetic and potentiated patient‐centered care. Patient‐centered care, is a philosophy that prioritises the needs, preferences, and values of patients in the delivery of healthcare services. It emphasises collaborative partnerships between healthcare providers and patients, fostering shared decision‐making and mutual respect (Araki 2019). The relationship between emotional labour behaviour and patient‐centered care is intertwined, as emotional labour influences the ability of nurses and nursing students to effectively provide patient‐centered care (Vinson and Underman 2020; Adler 2020). When healthcare professionals engage in deep acting, authentically experiencing and expressing emotions, they can establish genuine connections with patients and foster trust, which are essential components of patient‐centered care (Grandey 2000). By effectively managing their emotions and displaying empathy, nurses can create an environment that promotes patient engagement, active participation in care decisions, and improved patient outcomes (McCormack et al. 2011). Conversely, surface acting, which involves masking or suppressing true emotions, can hinder the provision of patient‐centered care. When nurses are emotionally detached or inauthentic in their interactions, patients may perceive a lack of empathy or caring, leading to decreased patient satisfaction and potentially compromised patient‐centered care (Schaubroeck and Jones 2000). The emotional strain resulting from surface acting may also contribute to emotional exhaustion and burnout among nurses, further impacting their ability to deliver patient‐centered care (Bakker et al. 2000).

Finally, the student nurses talked about how they were particularly honest with young and elderly clients and how they thought they were incompetent nurses when their patients passed away under the “genuine acting” topic. There are research papers that suggest that genuine acting was one of the most prevalent practices among students and professional nurses (Dempsey et al. 2023).

### 6.1 Limitations

Considering there are few research articles on the emotional labour behaviour of students of nursing, the present research explored the lived experiences of nursing students regarding their emotional labour behaviour and their encounters with managing feelings in healthcare settings, and added to the body of knowledge. However, one of the main limitations in this study is that it reflected the experiences of students at one university in Saudi Arabia. Therefore, this study's findings are restricted to the discussions and cannot be extrapolated. However, Lincoln and Guba (1986) contend that instead of reductive worries like sample size, generalisation of results from naturalistic investigations is connected with adaptability and applicability. It is suggested that these results have ramifications for other countries with comparable instructive procedures since they offer important new information into the practical learning encounters of student nurses in Saudi Arabia. In addition, feelings that clash with ethical standards give rise to emotional labour; thus students could have had trouble articulating this and be hesitant to share their full perspectives since it is a quite sensitive issue to address.

## 7 Conclusion

As a summary, it was found that students in nursing rendered attempts to control their facial expressions when speaking with patients so as not to show their feelings, that they occasionally felt like frauds, that emotional labour behaviour influenced their actions in practical situations, that a shortage of training and expertise was among the causes, and that an encouraging clinical atmosphere would influence how they handled their emotions. The ability to recognise emotions and respond naturally will likely play a significant role in offering high‐quality care to students who enter the field.

The findings of this study contribute to the existing literature by emphasising the significance of emotional labour behaviours in the context of patient‐centered care. This study extends the current knowledge by highlighting the potential negative impact of surface acting on patient‐centered care. When healthcare professionals engage in inauthentic emotional displays or emotional detachment, patients may perceive a lack of empathy, leading to decreased patient satisfaction and compromised patient‐centered care. It underscores the need for healthcare institutions to provide support and resources to help nurses and nursing students effectively manage their emotional labour, fostering a culture that prioritises patient‐centered care.

## 8 Implications for Practice and Research

The study emphasises how emotional labour behaviour is a common occurrence for nursing students throughout their practicum experience, which has an impact on how well they communicate with patients generally. In order to control the emotional labour at this time, instructors, nursing staff, and managers would play crucial responsibilities. Alongside receiving instruction on emotional management techniques and emotional labour behaviour, students' levels of stress and emotional labour behaviour should be assessed before training in order to address the issues brought on by this conduct. The nursing curricula should also incorporate emotional intelligence instructional courses to lessen emotional behaviour. The effectiveness and fulfilment of students' clinical experiences are both impacted by emotional labour behaviour, according to recent study. By creating a supportive therapeutic atmosphere and identifying the circumstances and causes that contribute to emotional labour, appropriate measures should be implemented. Seminars and conferences should be planned to achieve experience exchange, particularly those combining first and final year students as well as peer instructional procedures to be incorporated into the program. Surface behaviour that adversely affects health care and educational outcomes rises when professionals and instructors fail to offer students the assistance they need. To address these issues, staff nurses and training students should come together, objectives should be clarified, orienting information ought to be provided, and instructors and nurse practitioners should be made more aware of emotional labour behaviours and how to handle emotions.

Conducting longitudinal studies to examine the long‐term effects of emotional labour on nursing students' well‐being, professional development, and retention in the nursing profession is recommended. This research can shed light on the potential consequences of prolonged emotional labour, such as burnout, job satisfaction, and career progression. In addition, comparing emotional labour experiences and strategies among nursing students in different educational settings or healthcare systems can be valuable. This research can provide insights into how cultural, institutional, or educational factors influence emotional labour behaviours and patient‐centered care. Further research can also be done while collaborating with disciplines such as psychology, sociology, or communication studies to gain a more comprehensive understanding of emotional labour in nursing. This interdisciplinary approach can provide a broader perspective on the emotional dynamics and interactions within healthcare settings.

## Author Contributions

The manuscript has been conceptualised and designed by Eco‐r‐180, who wrote the proposal, reviewed it and developed the manuscript. Eco‐r‐180 worked on validation, data curation, analysis, writing and finalising the manuscript. Eco‐r‐180 also worked in data validation and analysis as well as reviewing the final draft and rewriting sections in the manuscript.

## Ethics Statement

The researcher was granted approval from the university's Research and Ethics Committee (Eco‐r‐180). All ethical considerations were applied according to the international Declaration of Helsinki's principles and guidelines, where the students were informed about all details of the study before recruitment and were not forced to be inducted. No disadvantages were reported to students who did not participate, and written informed consent was obtained.

## Consent

The author has nothing to report.

## Conflicts of Interest

The author declares no conflicts of interest.

## Data Availability

The data that support the findings of this study are available on request from the corresponding author. The data are not publicly available due to privacy or ethical restrictions.
