# Correction: Emotional Labour Behaviour of Nursing Students: A Phenomenological Qualitative Study

**DOI:** 10.1002/nop2.70546

**Published:** 2026-04-07

Al‐Osaimi, D. N. (2026). Emotional Labour Behaviour of Nursing Students: A Phenomenological Qualitative Study. *Nursing Open*, *13*(1), e70362.

In the ‘Acknowledgements’ section, the text ‘The researcher would like to acknowledge the support of Eco‐r‐180, Research supporting project number (ORF 2023R925), King Saud University, Riyadh, Saudi Arabia; Support Number, Eco‐r‐180’ was incorrect. This should have read: ‘Research supporting project number (ORF2024R925), King Saud University, Riyadh, Saudi Arabia’.

We apologize for this error.

